# LncRNA H19 contributes to hippocampal glial cell activation via JAK/STAT signaling in a rat model of temporal lobe epilepsy

**DOI:** 10.1186/s12974-018-1139-z

**Published:** 2018-04-10

**Authors:** Chun-Lei Han, Ming Ge, Yun-Peng Liu, Xue-Min Zhao, Kai-Liang Wang, Ning Chen, Wen-Jia Meng, Wei Hu, Jian-Guo Zhang, Liang Li, Fan-Gang Meng

**Affiliations:** 1 Department of Functional Neurosurgery, Beijing Neurosurgical Institute, Capital Medical University, Beijing, 100050 China; 2 Beijing Key Laboratory of Neuromodulation, Beijing Municipal Science and Technology Commission, Beijing, 100050 China; 3 Department of Neurosurgery, Beijing Children’s Hospital, Capital Medical University, Beijing, 100045 China; 4 Department of Neurosurgery, Beijing Tiantan Hospital, Capital Medical University, Beijing, 100050 China; 5 The Third Division of Clinical Medicine, China Medical University, Shenyang, 110122 Liaoning Province China; 6 Department of Neurology, University of Florida, Gainesville, Florida 32607 USA; 7 Department of Pathology, School of Basic Medical Sciences, Capital Medical University, No. 10 Xi TouTiao, You An Men Street, Beijing, 100069 China

**Keywords:** Temporal lobe epilepsy, lncRNA H19, Astrocytes, Microglia, Inflammatory response

## Abstract

**Background:**

Astrocyte and microglia activation are well-known features of temporal lobe epilepsy that may contribute to epileptogenesis. However, the mechanisms underlying glia activation are not well understood. Long non-coding RNA (lncRNA) H19 has diverse functions depending on physiological or pathological state, and its role in epilepsy is unknown. We previously demonstrated that H19 was significantly upregulated in the latent period of epilepsy and may be associated with cell proliferation and immune and inflammatory responses. We therefore speculated that H19 is involved in the hippocampal glial cell activation during epileptogenesis.

**Methods:**

H19 was overexpressed or knocked down using an adeno-associated viral vector delivery system. A rat status epilepticus model was induced by intra-amygdala kainic acid injection. Astrocyte and microglia activation were assessed by immunofluorescence and western blot analyses. Expression of proinflammatory cytokines and components of the Janus kinase (JAK)/signal transducer and activator of transcription (STAT) signaling pathways were evaluated with western blotting.

**Results:**

H19 overexpression induced the activation of astrocytes and microglia and the release of proinflammatory cytokines (interleukin-1β and interleukin-6 and tumor necrosis factor-α) in the hippocampus, whereas H19 knockdown inhibited status epilepticus-induced glial cell activation. Moreover, H19 activated JAK/STAT signaling by promoting the expression of Stat3 and c-Myc, which is thought to be involved in astrocyte activation.

**Conclusions:**

LncRNA H19 contributes to hippocampal glial cell activation via modulation of the JAK/STAT pathway and could be a therapeutic tool to prevent the development of epilepsy.

**Electronic supplementary material:**

The online version of this article (10.1186/s12974-018-1139-z) contains supplementary material, which is available to authorized users.

## Background

Temporal lobe epilepsy (TLE) is one of the most common types of intractable epilepsy and is characterized by the periodic and unpredictable occurrence of seizures. Glial cell activation and proliferation, a well-described pathological feature of TLE, can alter blood-brain barrier integrity and ion and neurotransmitter homeostasis and cause an inflammatory response, resulting in neuronal hyperexcitability and the generation and spread of seizure activity [1–3]. Although impairment of these functions is thought to be associated with the pathophysiology of epilepsy, the mechanisms underlying glial cell activation are complex and are not fully understood [1].

Long non-coding RNA (lncRNA) H19, an imprinted gene, is located on human chromosome 11 and is transcribed from the maternally inherited allele [4]. Despite being identified over 20 years ago, the function of H19 remains unclear and its pathological role as a non-coding RNA has only recently been elucidated [5]. H19 has diverse functions depending on physiological and pathological state. In the central nervous system (CNS), H19 is overexpressed in glioblastoma tissue and promotes the proliferation, differentiation, migration, and invasion of glioma cells [6, 7]. However, the biological function of H19 in non-neoplastic CNS diseases including epilepsy remains unknown.

We previously showed by high-throughput microarray and bioinformatics analyses that H19 is upregulated in the latent period of TLE in rat and is involved in various aspects of epileptogenesis, including cell proliferation and immune and inflammatory responses [8]. We therefore speculated that H19 may be involved in hippocampal glial cell activation during epileptogenesis. This was investigated in the present study by gain- and loss-of-function studies in a rat model of TLE. We also examined the possible downstream targets of H19.

## Methods

### Animal and human samples

Male Sprague–Dawley rats weighing 200–220 g were obtained from Vital River Experimental Animal Technology Co. (Beijing, China) and were housed in a temperature-controlled room with free access to standard food and water under a 12:12-h light/dark cycle. Surgically resected hippocampus specimens were obtained from patients with intractable TLE who underwent surgical treatment at Beijing Tiantan Hospital. Control hippocampal tissue was obtained from autopsies of four patients without a history of epilepsy or other neurological diseases within 8 h after death.

### H19 overexpression and knockdown

H19 was overexpressed or silencing using an adeno-associated viral (AAV) vector delivery system as previously described [8]. Briefly, a vector harboring H19 (AAV-H19) or a short hairpin RNA targeting H19 (AAV-shRNA) was constructed by Gene Chem Co. (Shanghai, China). The negative control was an empty AAV vector (AAV-NC) or one harboring a scrambled sequence (AAV-Scr: 5′-TTCTCCGAACGTGTCACGT-3′). The titers used were 1.0 × 10^12^ for AAV9-H19 and 4.0 × 10^12^ for AAV9-shRNA. A total of 6 μl AAV was infused into the right dorsal hippocampus (3.12 mm posterior to the bregma, 3.0 mm lateral to the midline, and 3.4 mm ventral to the bregma) and ventral hippocampus (5.04 mm posterior to the bregma, 5.0 mm lateral to the midline, and 6.4 mm ventral to the bregma; 3 μl at each location) [9] through a microsyringe at a speed of 0.2 μl/min.

### Epilepsy model

A kainic acid (KA)-induced status epilepticus (SE) model was established by intra-amygdala microinjection of KA 14 days after AAV injection according to our previously described technique [8]. Briefly, the rats were placed in a stereotaxic apparatus (David Kopf Instruments, Tujunga, CA, USA), and 0.7 μl KA (1 μg/μl; Sigma–Aldrich, St. Louis, MO, USA) was injected into the right amygdala (2.76 mm posterior to the bregma, 4.5 mm lateral to the midline, and 8.6 mm ventral to the bregma) [9] at a speed of 0.2 μl/min. Sham-operated controls were injected with an equal volume of saline.

### Immunofluorescence analysis

Coronal sections (25 μm) were prepared at the level of the dorsal hippocampus (2.50–3.50 mm posterior to the bregma). Frozen sections were dried, washed, permeabilized, blocked in 5% goat serum, and incubated overnight with antibodies against neuronal nuclei (NeuN) (ab177487, 1:500 and ab104224, 1:200), glial fibrillary acidic protein (GFAP) (ab7260, 1:500), and OX42 (ab1211, 1:100) (all from Abcam, Cambridge, MA, USA). Immunolabeled sections were washed and incubated with goat secondary antibodies conjugated with Alexa Fluor 594 or Alexa Fluor 488 (Merck Biosciences, Nottingham, UK). Sections were mounted with medium containing 4′,6-diamidino-2-phenylin-dole (DAPI) (Vector Laboratories, Burlingame, CA, USA), and images were captured using an inverted fluorescence microscope (Olympus, Tokyo, Japan). GFAP+ or OX42+ cells were manually counted using ImageJ software (US National Institutes of Health, Bethesda, MD, USA).

### Western blotting

Western blot analysis was performed as previously described [10] using the following primary antibodies: rabbit polyclonal anti-GFAP (ab7260, 1:1000), mouse monoclonal anti-OX42 (ab1211, 1:500), rabbit polyclonal anti-interleukin (IL)-1β (ab9722; 1:500), mouse monoclonal anti-IL-6 (ab9324; 1:500), rabbit polyclonal anti-tumor necrosis factor (TNF)-α (ab6671; 1:500), rabbit monoclonal anti-p-Stat3 (ab76315; 1:500), and rabbit polyclonal anti-c-Myc (ab39688; 1:500) (all from Abcam, Cambridge, MA, USA). Rabbit monoclonal anti-glyceraldehyde 3-phosphate dehydrogenase (GAPDH) antibody (Abcam, ab181602, 1:3000) was used as a control. Protein band density was quantified using an Epson V330 Photo scanner (Seiko Epson Co., Nagano, Japan) and Quantity One software (Bio-Rad, Hercules, CA, USA).

### Statistical analysis

Data are presented as mean ± standard error of the mean. Two-group comparisons were made with the unpaired Student’s *t* test, and multi-group comparisons were made by one-way analysis of variance followed by Sidak’s multiple comparison tests using Prism 5 software (GraphPad Inc., San Diego, CA, USA). Significance was accepted at *P* < 0.05.

## Results

### Astrocytes and microglia are activated in the hippocampus of epileptic rats

**Fig. 1:**
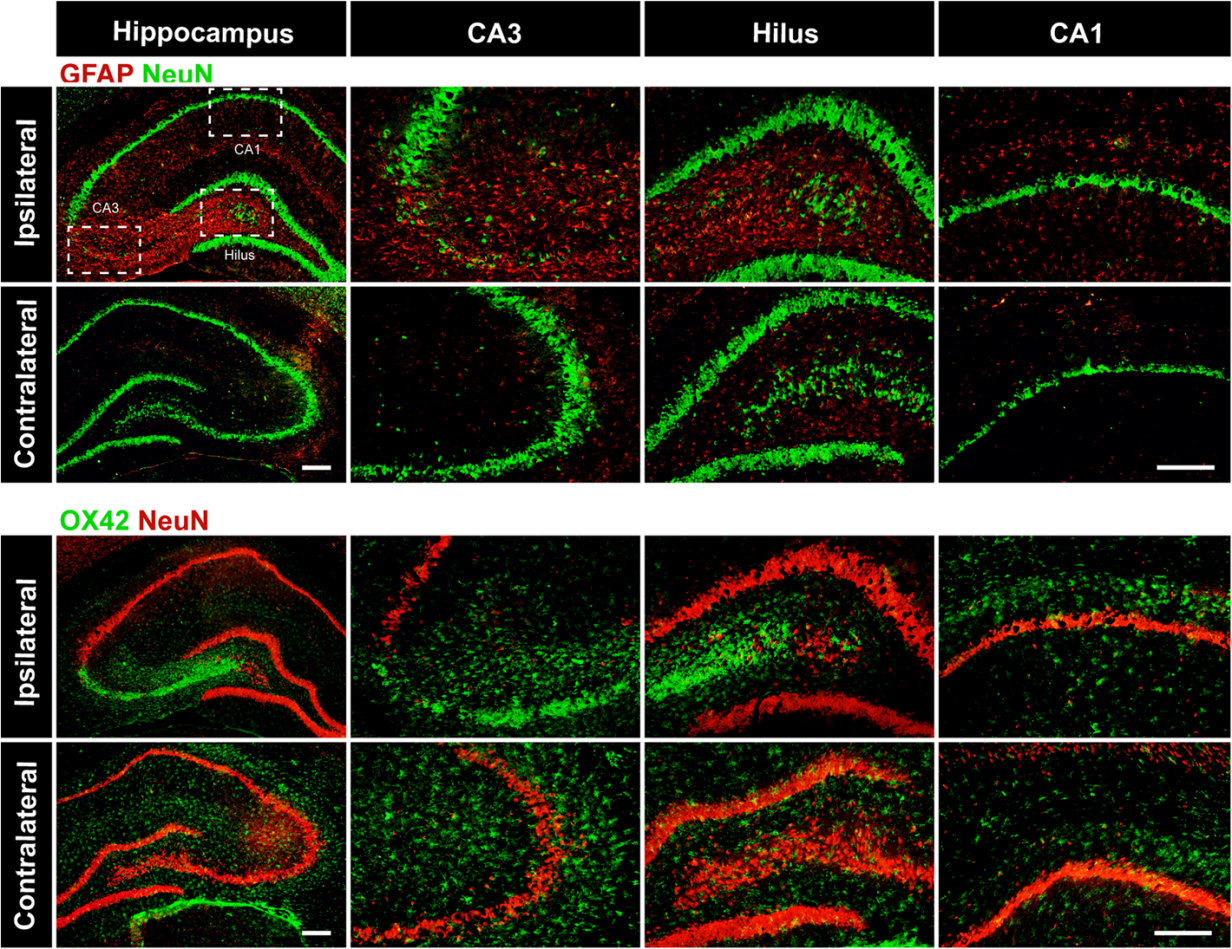
Astrocytes and microglia are activated in the hippocampus of epileptic rats. Representative fluorescence micrographs of GFAP and OX42 expression in the hippocampus of rats 30 days after SE. High-magnification images correspond to the labeled boxes in the left panels. Scale bar = 200 μm

**Fig. 2:**
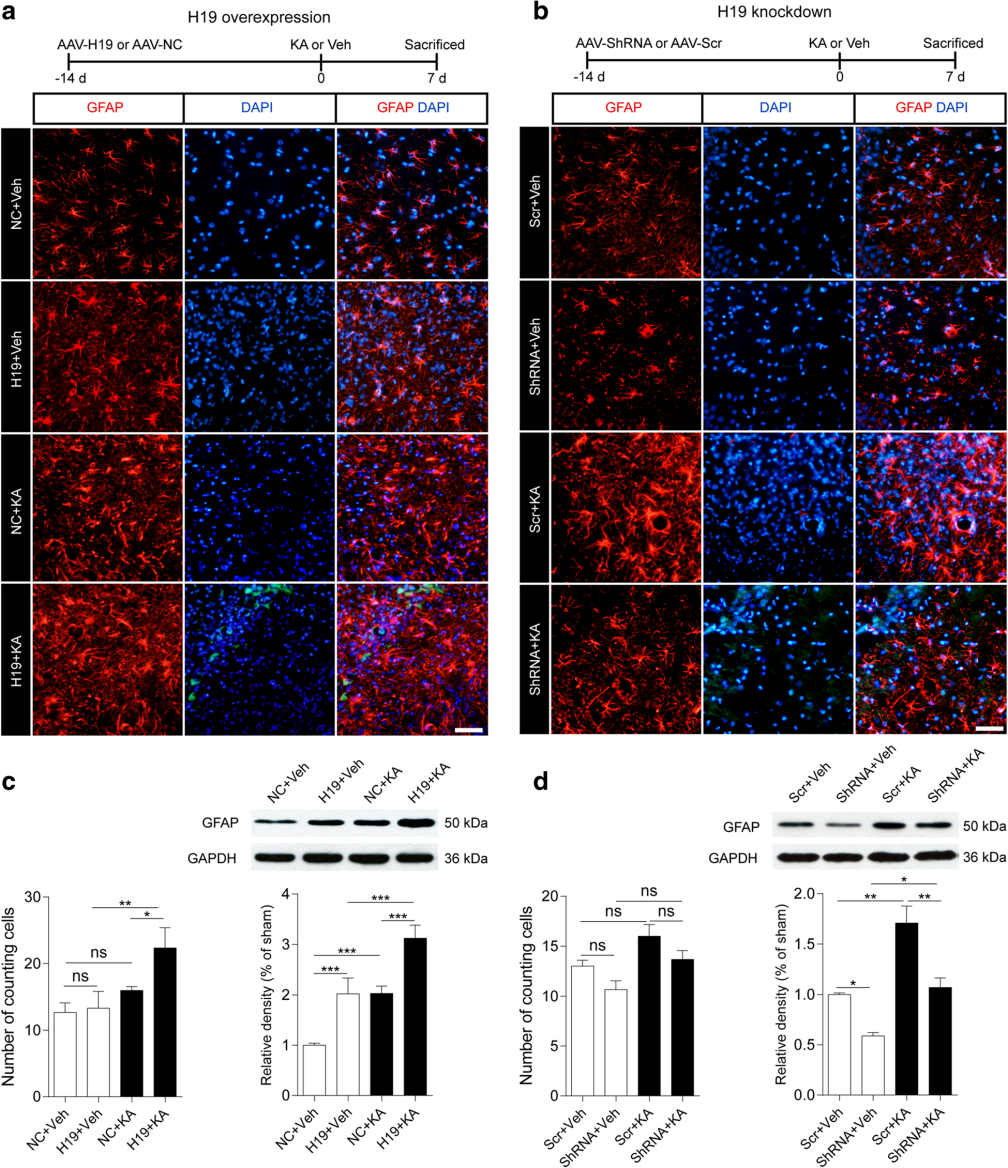
H19 is involved in astrocyte activation in the hippocampus of epileptic rats. **a**, **b** Top: experimental timeline. Middle: representative fluorescence micrographs of GFAP expression in the hippocampus of H19 overexpression (**a**) and H19 knockdown (**b**) rats with or without KA treatment for 7 days. Scale bar = 50 μm. Bottom panels show counts of cells in the CA3 regions of the hippocampus ipsilateral to the KA injection side (*n* = 3). **c**, **d** Western blot analysis of GFAP protein levels in the CA3 subfield of the hippocampus of H19 overexpression (**c**) and H19 knockdown (**d**) rats with or without KA treatment for 7 days (*n* = 3–4). Protein bands were quantified by densitometry and normalized to the level of GAPDH. Data represent mean ± SEM. *P < 0.05, **P < 0.01, ***P < 0.001. NC rats injected with empty AAV vectors, Scr rats injected with scrambled AAV vectors, ShRNA rats injected with AAV vectors containing short hairpin RNA targeting H19

**Fig. 3:**
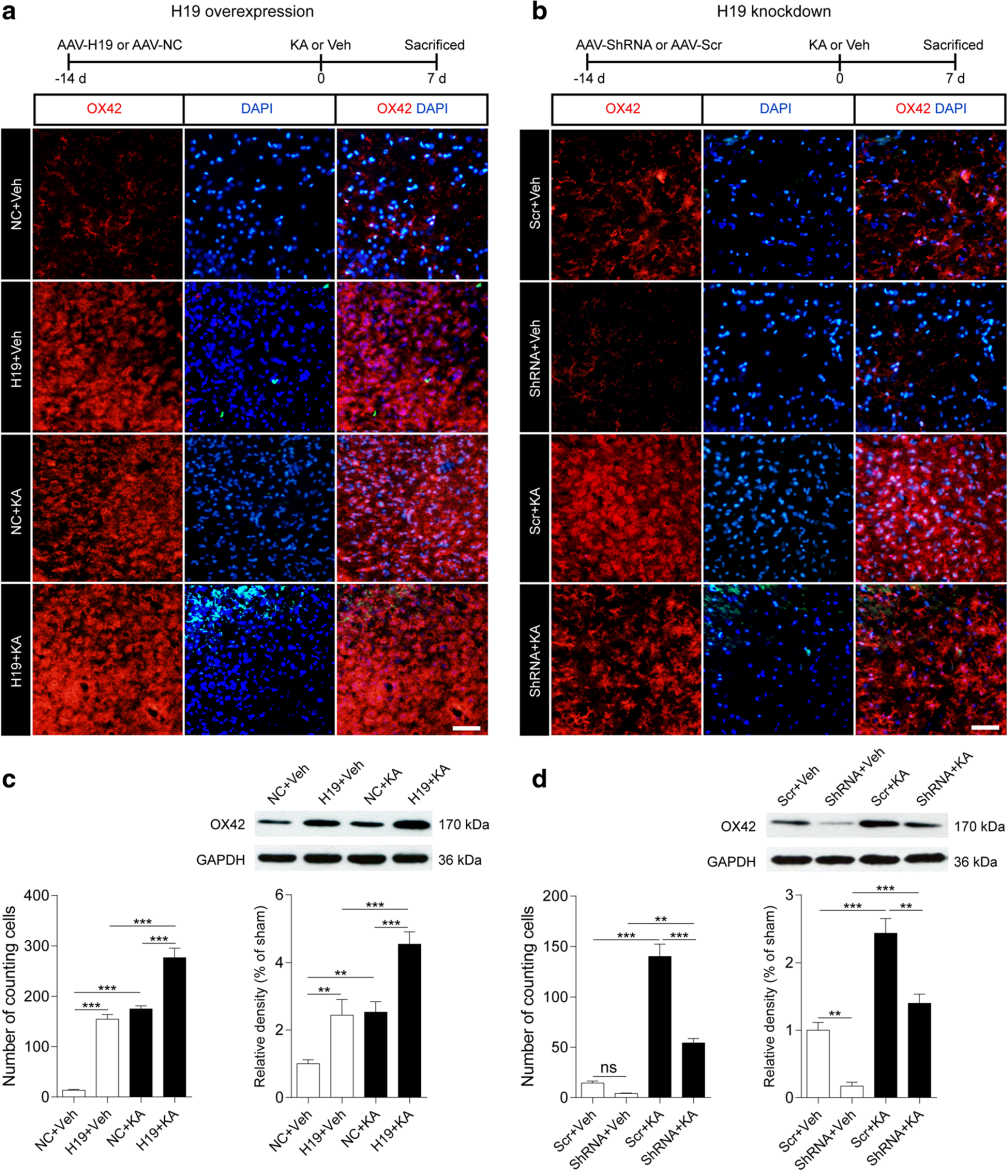
H19 is involved in the activation of microglia in the hippocampus of epileptic rats. **a**, **b** Top: experimental timeline. Middle: representative fluorescence micrographs of OX42 expression in the hippocampus of H19 overexpression (**a**) and H19 knockdown (**b**) rats with or without KA treatment for 7 days. Scale bar = 50 μm. Bottom panels show counts of cells in the CA3 regions of the hippocampus ipsilateral to the KA injection side (*n* = 3). **c**, **d** Western blot analysis of OX42 protein level in the CA3 subfield of the hippocampus of H19 overexpression (**c**) and H19 knockdown (**d**) rats with or without KA treatment for 7 days (*n* = 3–4). Protein bands were quantified by densitometry and normalized to the level of GAPDH. Data represent mean ± SEM. *P < 0.05, **P < 0.01, ***P < 0.001. NC rats injected with empty AAV vectors, Scr rats injected with scrambled AAV vectors, ShRNA rats injected with AAV vectors containing short hairpin RNA targeting H19

We first examined GFAP and OX42 expression in the hippocampus of epileptic rats by immunofluorescence analysis to evaluate astrocyte and microglia activation, respectively. GFAP and OX42 immunoreactivity was detected in the ipsilateral hippocampus 7 days (latent period) (Figs. 2 and 3) or 30 days (chronic period) (Fig. 1) after KA-induced SE. The number of GFAP+ cells was increased, and activated astrocytes showed hypertrophy with a large cytoplasm and thick processes. The number of OX42+ cells was also increased, and the morphology of the activated microglia changed from spindle shape to oval with thickened processes. Fewer activated glial cells were observed in the contralateral as compared in the ipsilateral hippocampus (Fig. 1).

### H19 is involved in the activation of astrocytes and microglia in the hippocampus of epileptic rats

Our previous study showed that H19 has diverse functions related to epileptogenesis [8] and is highly expressed in the seizure-free latent period of TLE. In the present study, we investigated the role of H19 in astrocyte and microglia activation by H19 overexpression and knockdown using an AAV delivery system [8]. Astrocyte and microglial activation were evaluated by GFAP and OX42 immunofluorescence and western blot analyses. Compared to the sham group (NC + Veh or Scr + Veh), KA-induced SE (KA + Veh) or H19 overexpression (H19 + Veh) alone induced the activation of astrocytes or microglia in the stratum radiatum of the hippocampal CA3 region (Figs. 2 and 3). Moreover, more activated cells were observed in the hippocampus of rats overexpressing H19 at 7 days after SE (H19 + KA). The observed SE-induced activation of astrocytes and microglia was partly inhibited by H19 knockdown (ShRNA + KA vs Scr + KA). A quantitative analysis of GFAP and OX42 protein levels in the hippocampal CA3 region at 7 days (Figs. 2 and 3) and 60 days (Additional file 1: Figure S1) after SE confirmed these results.

**Fig. 4:**
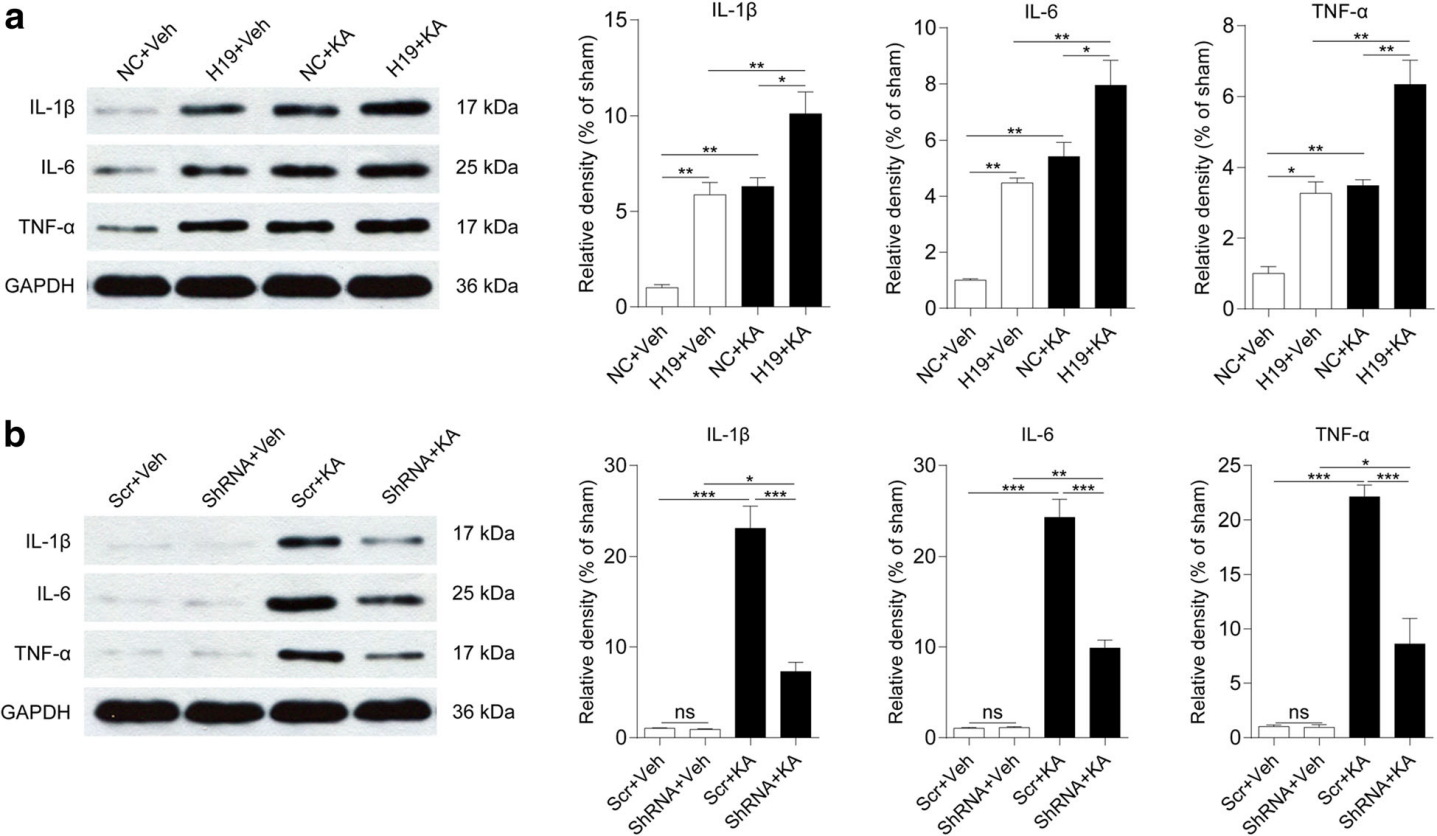
H19 induces pro-inflammatory cytokine expression. **a**, **b** Protein levels of IL-1β and IL-6 and TNF-α in the CA3 subfield of the hippocampus of H19 overexpression (**a**) or H19 knockdown (**b**) rats with or without KA treatment for 7 days (*n* = 3–4). Protein bands were quantified by densitometry and normalized to the level of GAPDH. Data represent mean ± SEM. *P < 0.05, **P < 0.01, ***P < 0.001. NC rats injected with empty AAV vectors, Scr rats injected with scrambled AAV vectors, ShRNA rats injected with AAV vectors containing short hairpin RNA targeting H19

We also examined proinflammatory cytokines released from the activated glia. H19 overexpression and KA-induced SE both stimulated the release of IL-1β and IL-6 and TNF-α in the CA3 subfield of the hippocampus (Fig. 4a). Cytokine release was further increased by H19 at 7 days after SE (Fig. 4a). H19 knockdown prevented the SE-induced increase in IL-1β and IL-6 and TNF-α levels (Fig. 4b). These results indicate that H19 plays an important role in astrocyte and microglia activation during epileptogenesis.

### H19 induces astrocyte and microglia activation via JAK/STAT signaling

**Fig. 5:**
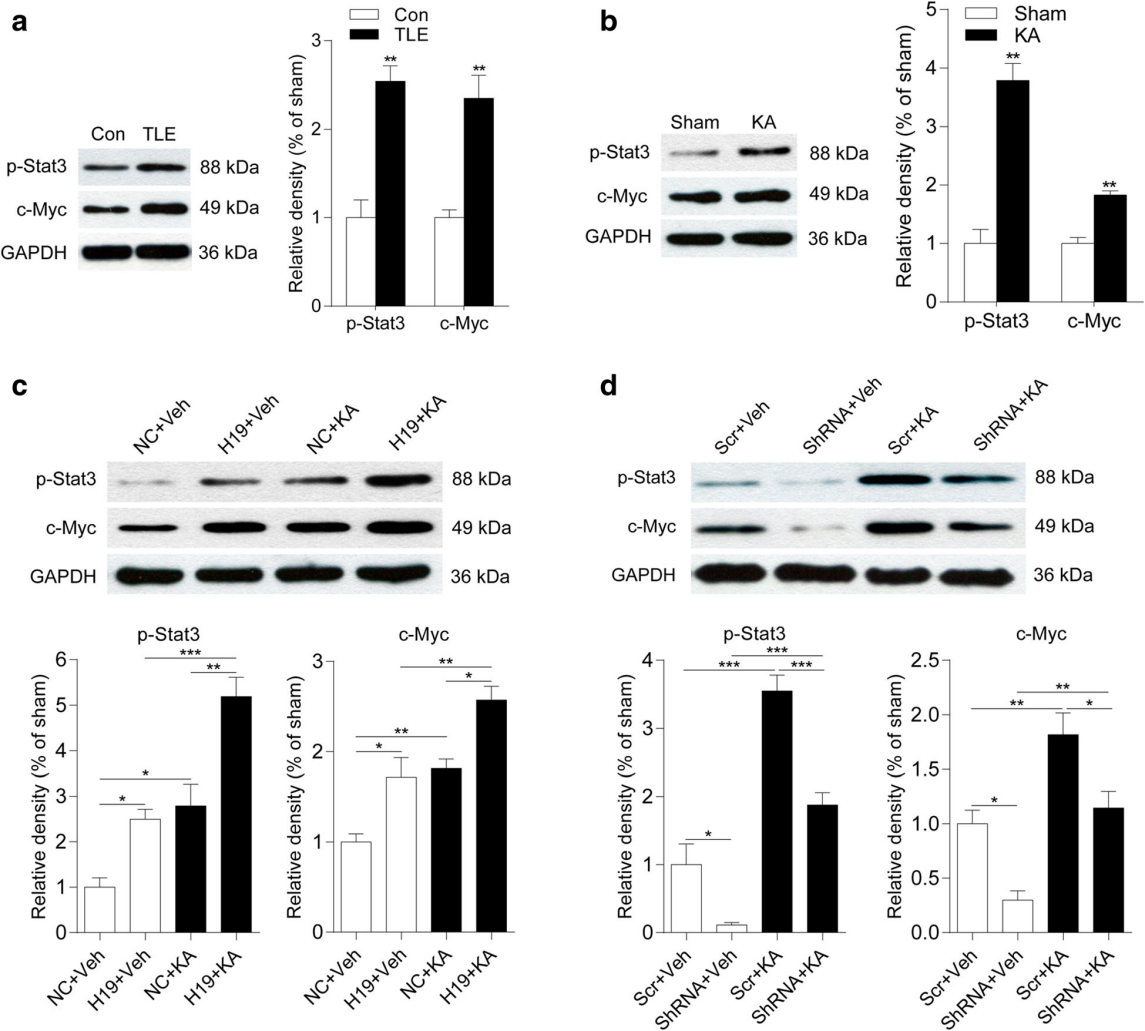
H19 stimulates p-Stat3 and c-Myc expression. **a**, **b** Protein levels of p-Stat3 and c-Myc in the hippocampal tissue samples from patients with TLE (*n* = 4) (**a**) and the hippocampus of epileptic rats 7 days after KA injection (*n* = 3) (**b**), as determined by western blotting. **c**, **d** Quantification of p-Stat3 and c-Myc protein levels in the CA3 subfield of the hippocampus of H19 overexpression (**c**) and H19 knockdown (**d**) rats with or without KA treatment for 7 days (*n* = 3–4). Protein bands were quantified by densitometry and normalized to the level of GAPDH. Data represent mean ± SEM. *P < 0.05, **P < 0.01, ***P < 0.001. NC rats injected with empty AAV vectors, Scr rats injected with scrambled AAV vectors, ShRNA rats injected with AAV vectors containing short hairpin RNA targeting H19

Stat3 plays a key role in astrocyte proliferation after central nervous system injury [11, 12] or SE [13]. We found here that the levels of phosphorylated Stat3 (p-Stat3) and its downstream effector c-Myc were upregulated in hippocampal tissue samples from patients with TLE (Fig. 5a) and from rats 7 days after SE (Fig. 5b) relative to the respective control samples, as determined with western blotting, suggesting that Janus kinase (JAK)/signal transducer and activator of transcription (STAT) signaling is involved in the activation of glial cells after SE. H19 overexpression alone increased p-Stat3 and c-Myc protein levels in the CA3 subfield of the hippocampus (Fig. 5c). H19 exacerbated these protein expressions in the rats at 7 days (Fig. 5c) and 60 days (Additional file 2: Figure S2A) after SE. On the contrary, H19 knockdown abolished the SE-induced increase in p-Stat3 and c-Myc in rats at 7 days (Fig. 5d) and 60 days (Additional file 2: Figure S2B) after SE. These results indicate that H19 promotes astrocyte and microglial activation via the JAK/STAT signaling pathway.

## Discussion

Most previous studies on H19 function focused on tumorigenesis. H19 was initially proposed as a tumor suppressor due to its capacity to suppress clonogenicity and tumorigenicity in tumor cells [14, 15]. However, recent studies showed that H19 acts as an oncogene by promoting cell proliferation, migration, invasion, and metastasis in various malignancies including glioblastoma [16–18]. Apart from these functions, H19 is also implicated in several other physiological conditions or diseases, such as cartilage degeneration in osteoarthritis [19], skeletal muscle differentiation and regeneration [20], and glucose metabolism in muscle cells [21]. However, the role of H19 in non-neoplastic CNS diseases including epilepsy remains unclear. In the present study, we provide the first evidence that H19 promotes glial cell activation and stimulates inflammation in the hippocampus of rats with TLE.

Astrocyte activation is a continuum that includes changes in molecular expression, progressive cellular hypertrophy, and, in severe cases, proliferation and scar formation [22]. In mild or moderate astrocyte activation, GFAP expression is slightly upregulated and the cell body and processes undergo hypertrophy, with little or no astrocyte proliferation. However, in severe diffuse reactive astrogliosis, GFAP expression is markedly increased, which is accompanied by extensive hypertrophy of the cell body and processes and astrocyte proliferation [23]. In the present study, astrocytes in the hippocampus of epileptic rats were activated in the latent and chronic phases of TLE, which is consistent with previous reports [13, 24]. Mild to moderate gliosis, which typically does not cause astrocyte proliferation, is usually observed in the early stages after SE [25]. We also found that astrocyte activation was mild or moderate, as evidenced by the upregulation of GFAP expression and cellular hypertrophy without an obvious increase in cell number in the latent period of TLE (7 days after SE). Furthermore, H19 induced an increase in GFAP expression and hypertrophy of astrocytes rather than cell proliferation in the latent period of SE. In the chronic period (30 days after SE), astrocyte activation was extreme with severe hypertrophy of the cell body and processes; moreover, astrocyte proliferation in areas of pyramidal neuron loss was comparable to the hippocampal sclerosis observed in TLE patients [26]. Unlike astrocytes, high expression of OX42 protein and cellular hypertrophy as well as proliferation of microglia was observed in both the latent and chronic periods of TLE, as previously reported [27]. Furthermore, H19 induced the upregulation of OX42 and cellular hypertrophy and increased the number of microglia in the latent period of SE. Recent studies have shown that inflammatory cytokines are produced both by microglia and astrocytes [28]; the increased levels of proinflammatory cytokines in epileptic rats observed here is in agreement with these findings. Moreover, H19 stimulated the release of proinflammatory cytokines. However, compared to sham rats, H19 knockdown did not inhibit proinflammatory cytokine expression, possibly because under normal conditions, proinflammatory cytokine levels in the hippocampus are too low to result in an observable difference upon H19 knockdown.

Molecular triggers that lead to glial cell activation and proliferation have not been fully characterized. There is increasing evidence to suggest that H19 has a growth-promoting function, since it enhances cell proliferation in tumors [29, 30] and other diseases [20, 31]. In the present study, we showed that H19 promotes astrocyte and microglia activation and proliferation under both epileptic and normal conditions. This is consistent with earlier reports as well as with our previous research [8]. Various intracellular signaling pathways associated with Stat3, nuclear factor κB, and nuclear respiratory factor (Nrf) mediate cell hypertrophy, proliferation, and pro- or anti-inflammatory effects in astrocytes [23]. The transcription factor Stat3, a key component of the JAK/STAT pathway, is important for astrocyte proliferation in CNS diseases [11, 32, 33]. P-Stat3 is highly expressed in the rat hippocampus during different phases of epilepsy and in the temporal lobe of TLE patients [13]. Astrocyte activation can be suppressed by inhibiting JAK/STAT signaling, indicating that Stat3 activation induces GFAP expression [13]. In our study, Stat3 as well as its downstream effector c-Myc in the JAK/STAT pathway were upregulated after SE in the rat hippocampus and in TLE patients. Furthermore, H19 overexpression induced whereas its knockdown inhibited Stat3 and c-Myc protein expression in both normal and epileptic rats. Thus, H19 can itself promote gliosis via JAK/STAT signaling in addition to its role in astrocyte activation.

## Conclusions

In summary, lncRNA H19 contributes to the activation of hippocampal astrocytes and microglia, as well as to the inflammatory response in epileptic rats. Furthermore, H19 may promote glial cell activation through the JAK/STAT pathway. Our findings reveal a novel lncRNA H19-mediated mechanism in seizure-induced glial cell activation and provide a basis for developing lncRNA-based strategies to prevent the development of epilepsy.

## Additional files

Additional file 1: H19 promotes GFAP and OX42 expression. (a, b) Western blot analysis of GFAP and OX42 protein levels in the CA3 subfield of the hippocampus of H19 overexpression (a) or H19 knockdown (b) rats with or without KA treatment for 60 days (*n* = 3–4). Protein bands were quantified by densitometry and normalized to GAPDH level. Data represent mean ± SEM. *P < 0.05, **P < 0.01, ***P < 0.001. NC, rats injected with empty AAV vectors; Scr, rats injected with scrambled AAV vectors; ShRNA, rats injected with AAV vectors containing short hairpin RNA targeting H19. (TIFF 469 kb)

Additional file 2: H19 promotes p-Stat3 and c-Myc expression. (a, b) Western blot analysis of p-Stat3 and c-Myc protein levels in the CA3 subfield of hippocampus of H19 overexpression (a) or H19 knockdown (b) rats with or without KA treatment for 60 days (*n* = 3–4). Protein bands were quantified by densitometry and normalized to GAPDH level. Data represent mean ± SEM. *P < 0.05, **P < 0.01, ***P < 0.001. NC, rats injected with empty AAV vectors; Scr, rats injected with scrambled AAV vectors; ShRNA, rats injected with AAV vectors containing short hairpin RNA targeting H19. (TIFF 454 kb)

## Abbreviations

AAV: Adeno-associated viral
CNS: Central nervous system
DAPI: 4′,6-diamidino-2-phenylin-dole
GAPDH: Glyceraldehyde 3-phosphate dehydrogenase
GFAP: Glial fibrillary acidic protein
IL: Interleukin
JAK: Janus kinase
KA: Kainic acid
lncRNA: Long non-coding RNA
NC: Negative control
NeuN: Neuronal nuclei
Nrf: Nuclear respiratory factor
p-Stat3: Phosphorylated Stat3
SE: Status epilepticus
shRNA: Short hairpin RNA
STAT: Signal transducer and activator of transcription
TLE: Temporal lobe epilepsy
TNF: Tumor necrosis factor
