# Supplementary figures and images for: LncRNA H19 contributes to hippocampal glial cell activation via JAK/STAT signaling in a rat model of temporal lobe epilepsy
Source: J Neuroinflammation. 2018 Apr 10;15:103. doi: 10.1186/s12974-018-1139-z (PMC5894243; doi:10.1186/s12974-018-1139-z)

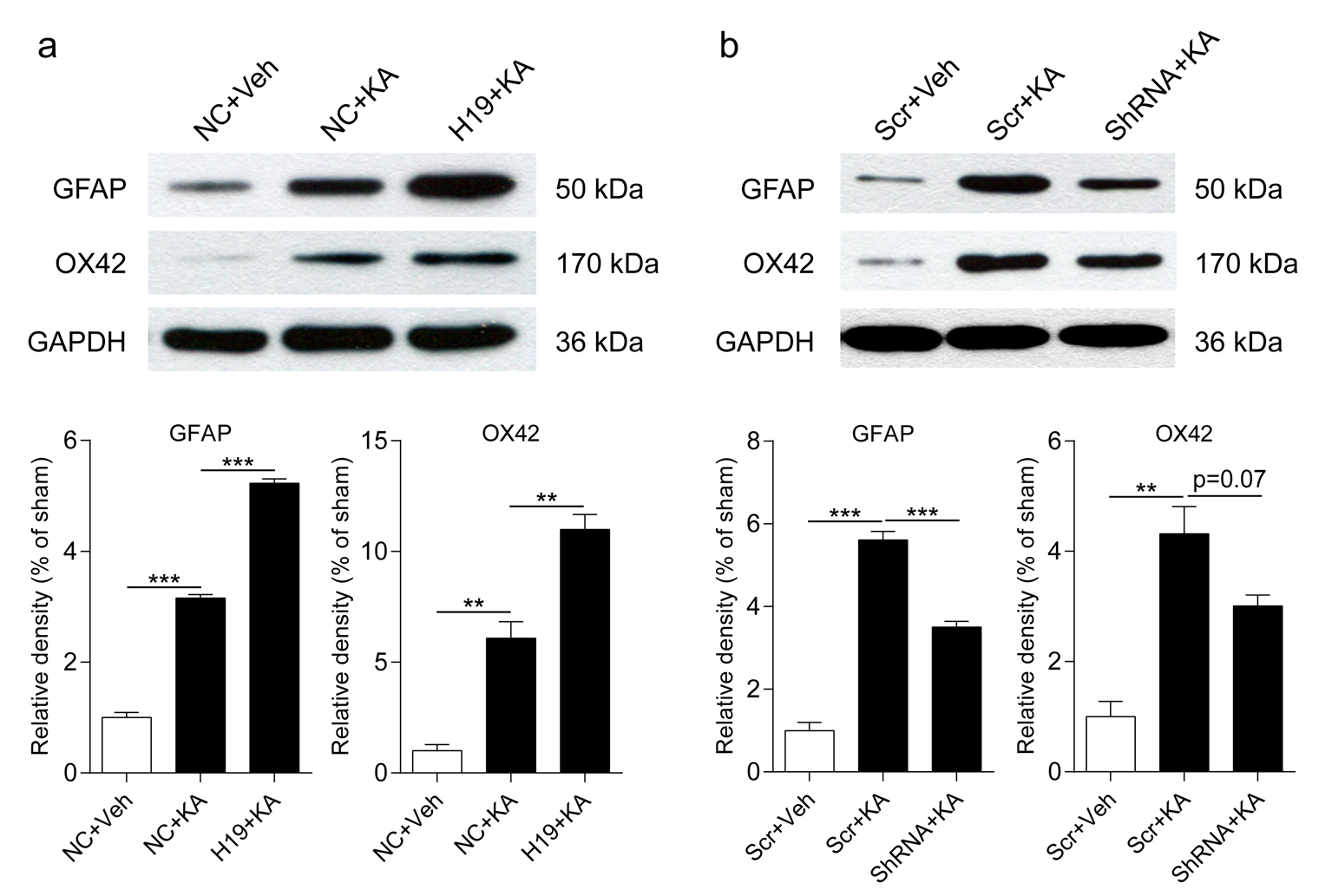

Supplement: Supplementary file 1 — H19 promotes GFAP and OX42 expression. (a, b) Western blot analysis of GFAP and OX42 protein levels in the CA3 subfield of the hippocampus of H19 overexpression (a) or H19 knockdown (b) rats with or without KA treatment for 60 days (n = 3–4). Protein bands were quantified by densitometry and normalized to GAPDH level. Data represent mean ± SEM. *P < 0.05, **P < 0.01, ***P < 0.001. NC, rats injected with empty AAV vectors; Scr, rats injected with scrambled AAV vectors; ShRNA, rats injected with AAV vectors containing short hairpin RNA targeting H19. (TIFF 469 kb) [file 12974_2018_1139_MOESM1_ESM.tif]

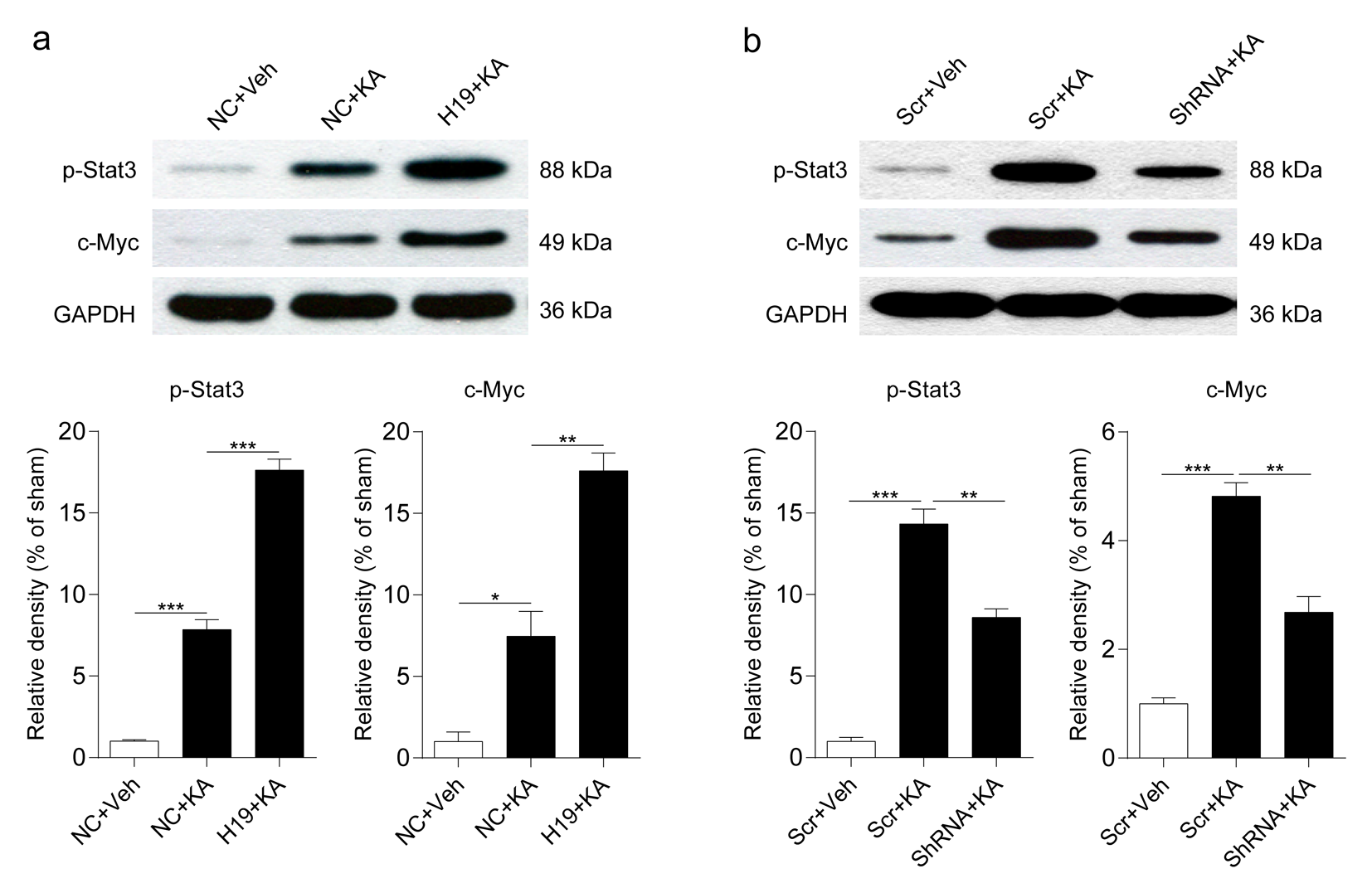

Supplement: Supplementary file 2 — H19 promotes p-Stat3 and c-Myc expression. (a, b) Western blot analysis of p-Stat3 and c-Myc protein levels in the CA3 subfield of hippocampus of H19 overexpression (a) or H19 knockdown (b) rats with or without KA treatment for 60 days (n = 3–4). Protein bands were quantified by densitometry and normalized to GAPDH level. Data represent mean ± SEM. *P < 0.05, **P < 0.01, ***P < 0.001. NC, rats injected with empty AAV vectors; Scr, rats injected with scrambled AAV vectors; ShRNA, rats injected with AAV vectors containing short hairpin RNA targeting H19. (TIFF 454 kb) [file 12974_2018_1139_MOESM2_ESM.tif]
